# Cerebellar Transcranial Direct Current Stimulation Improves Maximum Isometric Force Production during Isometric Barbell Squats

**DOI:** 10.3390/brainsci10040235

**Published:** 2020-04-14

**Authors:** Rouven Kenville, Tom Maudrich, Dennis Maudrich, Arno Villringer, Patrick Ragert

**Affiliations:** 1 Institute for General Kinesiology and Exercise Science, Faculty of Sport Science, University of Leipzig, D-04109 Leipzig, Germany; maudrich@cbs.mpg.de (T.M.); patrick.ragert@uni-leipzig.de (P.R.); 2 Department of Neurology, Max Planck Institute for Human Cognitive and Brain Sciences, D-04103 Leipzig, Germany; dmaudrich@cbs.mpg.de (D.M.); villringer@cbs.mpg.de (A.V.); 3 Clinic for Cognitive Neurology, University of Leipzig, 04103 Leipzig, Germany; 4 MindBrainBody Institute at Berlin School of Mind and Brain, Charité-Universitätsmedizin Berlin and Humboldt-Universität zu Berlin, 10099 Berlin, Germany

**Keywords:** transcranial direct current stimulation (tDCS), whole-body movement, motor system, muscle strength

## Abstract

Maximum voluntary contraction force (MVC) is an important predictor of athletic performance as well as physical fitness throughout life. Many everyday life activities involve multi-joint or whole-body movements that are determined in part through optimized muscle strength. Transcranial direct current stimulation (tDCS) has been reported to enhance muscle strength parameters in single-joint movements after its application to motor cortical areas, although tDCS effects on maximum isometric voluntary contraction force (MIVC) in compound movements remain to be investigated. Here, we tested whether anodal tDCS and/or sham stimulation over primary motor cortex (M1) and cerebellum (CB) improves MIVC during isometric barbell squats (iBS). Our results provide novel evidence that CB stimulation enhances MIVC during iBS. Although this indicates that parameters relating to muscle strength can be modulated through anodal tDCS of the cerebellum, our results serve as an initial reference point and need to be extended. Therefore, further studies are necessary to expand knowledge in this area of research through the inclusion of different tDCS paradigms, for example investigating dynamic barbell squats, as well as testing other whole-body movements.

## 1. Introduction

Muscle strength not only predicts athletic performance and/or efficacy but also contributes to the accomplishment of various tasks throughout everyday life, for example, walking, climbing stairs, and running [1,2,3]. Core features of muscle strength, such as maximum isometric voluntary contraction force (MIVC), depend on central nervous control of the number of active motor units and their firing rate. To date, central mechanisms of muscle strength remain insufficiently explored, especially regarding potential differentiations of the neuronal sites responsible for muscle strength regulation between single-joint (e.g., finger-pinch) and whole-body movements (e.g., squats). 

Previous neuroimaging studies showed both primary motor cortex (M1) and cerebellum (CB) to change their activity with different force requirements in single-joint movements [4,5,6,7], although it remains to be fully understood which cortical/subcortical structures concur to enable muscle strength and control in whole-body movements, i.e., movements that require orchestrated interplay between multiple joints and muscles. Since most neuroimaging methods are not ideally suited to investigating whole-body movements, neuromodulatory approaches have been employed to uncover strength related brain-muscle associations [8]. 

Transcranial direct current stimulation (tDCS) is a non-invasive procedure, which stimulates brain regions applying weak direct currents through the skull. Depending on the polarity, stimulated regions either show enhanced or decreased excitability. tDCS has been used to uncover neural links between muscle strength and related brain areas through modulating their excitability. Accordingly, numerous studies have demonstrated tDCS related increases in muscle strength endurance [9,10,11] and MIVC [12,13,14], particularly for upper extremities. Lower extremity muscle strength modulation has also been assessed through tDCS application, although results are rather inconclusive. For example, some results showed tDCS to be effective in modulating isometric muscle strength [10,14,15], whereas other studies provide evidence to the contrary [16,17,18]. It is of note that, among others stimulation sites, current density (mA/cm^2^), as well as strength training background of participants all differ between studies, making interpretations difficult. Nevertheless, a common ground regarding applicable tDCS protocols can be isolated. As such, most studies examining tDCS–MIVC modulations have stimulated M1, used a current intensity of 2 mA and stimulated for 20 min [8]. Additionally, enhancing effects of tDCS on MIVC have solely been reported for anodal tDCS, although cathodal effects have been examined [9,14], which may be rooted in increased cortical excitability and cross-activation, as well as reduced short-interval intracortical inhibition (SICI) [8,19,20]. 

TDCS induced modulations of MIVC remain to be investigated in whole-body tasks. During such tasks, potential target areas expand beyond motor cortical regions, as it is known that, apart from M1, SMA, and PMC, the cerebellum plays an essential part in motor control of whole-body movements, especially in movements that require appropriate and continuous postural control, e.g., squats and walking [21]. Here, the cerebellum has been shown to support postural control responses by adapting motor actions concerning specific task requirements [22,23]. Patients suffering from cerebellar pathologies (e.g., dysmetria and cerebellar ataxia) exhibit severe restrictions relating to control of kinematic and dynamic movement parameters during whole-body movement execution [24,25,26]. Selective studies even report of individual participants being able to jump on one leg, yet unable to coordinate both legs simultaneously after splitting of the cerebellar vermis [27]. Interestingly, in animal studies it could be shown that cerebellar discharge precedes cortical discharge during whole-body movement execution [28], hinting at a temporal hierarchy between both structures favoring the cerebellum. Further, in monkeys, the ability to execute unconstrained movements was greatly impaired during partial cerebellar inactivation when compared to constrained movement execution [29]. Accordingly, different aspects of whole-body movements have been modulated through cerebellar tDCS (CB-tDCS), which relies on the same principles as conventional tDCS. Few studies have examined CB-tDCS effects in compound or whole-body movements. For example, adaptations during a split-belt treadmill walking task were greatest for anodal CB-tDCS in healthy subjects when compared to cathodal and sham conditions [30]. CB-tDCS seems to be most effective (compared to M1-tDCS and sham stimulation (SH-tDCS)) regarding improvements in visuomotor tasks. Additionally, it was demonstrated that CB-tDCS elicits improvements in postural control, both in healthy participants [31] as well as chronic stroke patients [32]. Nevertheless, some studies show no effects of CB-tDCS on balance control in healthy participants [32,33]. To date, no study has tested the effects of CB-tDCS on core features of muscle strength, such as MIVC. 

In summary, although the physiological effects of both M1- and CB-tDCS remain to be fully understood, both stimulation sites have been shown to affect motor function. Specifically, M1-tDCS was able to increase muscle strength parameters such as MIVC in some instances, while CB-tDCS proved to be behaviorally beneficial regarding whole-body movement components such as postural control. Based on the aforementioned findings, we here hypothesized that tDCS over M1 and CB is capable of evoking increases in MIVC during isometric barbell squats (iBS) as compared to sham stimulation.

## 2. Materials and Methods

### 2.1. Participants

The study was approved by the local ethics committee of the Medical Faculty at the University of Leipzig (ref.-nr. 034/17-ek) and all participants gave their written informed consent to participate in the experiments per the Declaration of Helsinki. Participants were excluded from the present study if the following exclusion criteria were present: neurological/psychological disease, intake of centrally acting drugs, caffeine or alcohol intake 24 h before the experiment, acute, chronic, and/or inadequately regenerated pathologies of the knee joint, the ankle joints, and/or the spine to minimize the risk of injury. Also, participants with regular practice of musical instruments and sports (>3 ^hrs^/_week_) were excluded from participation in this study. This was motivated by the fact that recent studies have shown that musical training induces functional and structural plasticity in the brain [34,35]. Furthermore, we intended to test participants without any background in organized strength training to omit results related to athletic expertise. 

Initially, we performed a sample size estimation (G*Power 3.1) [36,37] based on previous results of tDCS induced modulation of MIVC in lower extremities [13,14] using the following parameters: for test family = *F*-test and statistical test = analysis of variance (ANOVA), a power value (probability of correctly rejecting a false null hypothesis) of 0.8 was chosen given a type I error rate of α= 5%. Additionally, the effect size (f) was set to 0.4, as previous related studies reported values in this range [13,14,18]. The estimated minimum sample size to obtain sufficient test power was *n* = 10. A total number of 25 healthy male (*n* = 13) and female (*n* = 12) participants (age: 23.29 ± 3.66 years (mean ± SD)) were enrolled in the present study. All participants were right-handed, as assessed by the Edinburgh Handedness Inventory with a laterality quotient (LQ) score of 91.98 ± 10.32. Due to incorrect measurements, 4 participants were excluded from further analyses. Additionally, one participant was excluded due to a neck injury during testing. All analyses were performed with the remaining 20 participants (age: 24.00 ± 3.65 years; LQ score: 91.34 ± 10.76).

### 2.2. Procedure

**Figure 1:**
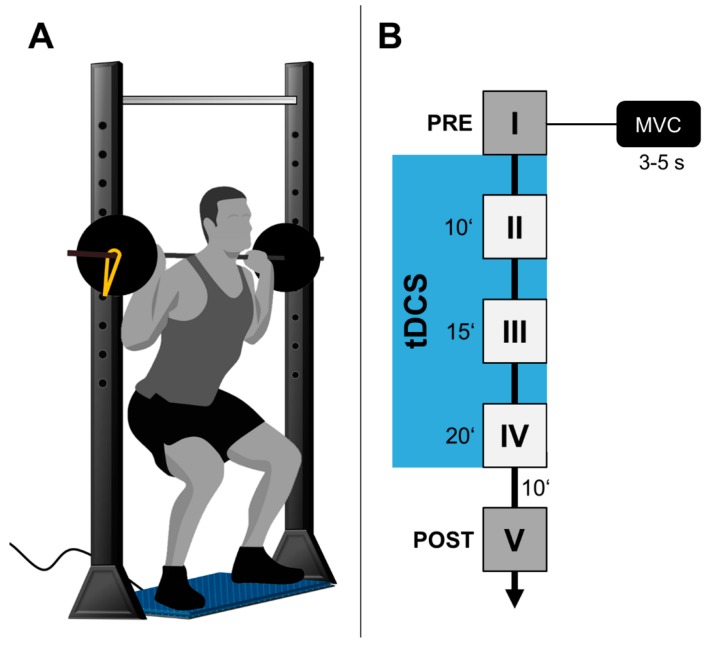
Study design. (**A**) Schematic representation of default positioning during isometric barbell squats (iBS) measurements. (**B**) Overview, regarding study procedures. Illustrated are all five maximum isometric voluntary contraction (MIVC) measurement blocks (I–V) alongside an exemplary depiction of a MIVC measurement conducted per one block for block I. MIVC blocks during stimulation are framed by a blue rectangle.

The experiment consisted of a randomized, counter-balanced, sham-controlled, double-blinded cross-over design where each participant performed during 3 experimental sessions, separated by at least 5 d, to prevent task-related impacts of cognitive and/or muscular fatigue. One researcher randomly assigned participants to CB-tDCS, M1-tDCS, or SH-tDCS using consecutive randomization. This researcher was uninvolved during subsequent data recording. All researchers conducting the experiment were unaware of the stimulation type. All participants performed a behavioral task of the lower extremities on three separate days using a different stimulation type (CB-tDCS, M1-tDCS, or SH-tDCS) for each session. A different tDCS-arrangement was randomly applied for each session to stimulate different brain regions during task performance for 20 min (for details see Figure 1). All participants were naïve in the iBS.

### 2.3. Behavioral Task (iBS)

At the beginning of each experimental session, instructions were given, focusing on the correct execution of the iBS. Participants were instructed to plant their feet and exert force without raising their heels during the performance of iBS. Additionally, each participant was instructed to keep a slight lumbar lordosis during iBS. Initial instructions were followed by a brief (3 min) warm-up program comprising of supervised executions of dynamic squats without additional weight and focusing on the aforementioned key aspects of correct movement execution (A, planting of the feet, B, slight lumbar lordosis). Before the first experimental session, the barbell position, corresponding to a 95° knee angle, was determined for each participant by using a digital protractor. Before baseline MIVC measurements were carried out, participants practiced the task for familiarization. 

Each session consisted of five blocks of iBS (before, 10, 15, and 20 min after stimulation onset and 10 min after stimulation termination (POST)). The duration to complete one block of iBS was approximately 30 s. The stimulation was started right after the last MIVC-measurement of the first block (before).

Maximum iBS-force (Newton (N)) was assessed using a multi-component force plate (Kistler type 9286AA, Kistler AG, Winterthur, Switzerland). Data were recorded with a sampling rate of 500 Hz. The force plate was placed in a straight vertical line below a fixed barbell mounted on a squat half rack (Barbarian-Line® Profi Half Rack, IFS GmbH, Wassenberg, Germany). For iBS, participants were instructed to step onto the force plate and under the standard barbell. The feet were placed and aligned along two marked lines on the surface of the force plate to standardize the position of the feet on the force plate. Lastly, shoulders were pressed against the fixed barbell and both hands grabbed the barbell while shoulders were slightly adducted. This position was assumed for all measurements. For MIVC measurements during the performance of iBS, all participants were told to push against the immovable barbell as hard as possible for 3–5 s. Peak force values of each MIVC-measurement were taken for the MIVC value of the respective block. Subjects rested in a seated position on a stool in between MIVC measurements. During rest phases, movements of the lower limbs were prohibited to avoid differences in excitability between participants. No feedback regarding iBS-MIVC-performance was given.

### 2.4. Transcranial Direct Current Stimulation

A tDCS current of 2 mA was delivered for 20 min (excluding 2 × 30 s of up- and down-ramping before and after stimulation respectively) using a battery-driven stimulator (neuroConn direct current (NC-DC)-stimulator; neuroConn GmbH, Ilmenau, Germany) and a pair of surface-soaked sponge electrodes. All tDCS conditions (M1-tDCS, CB-tDCS, SH-tDCS) were randomly assigned within and counter-balanced across participants. The anode (35 cm^2^, current density: 0.057 mA/cm^2^) was placed either over the bilateral M1 leg area or the bilateral cerebellum, with the cathode (reference; 100 cm^2^, current density 0.020 mA/cm^2^) placed on the medial part of the supraorbital bone (tDCS of bilateral M1 leg area) or the right musculus buccinator (tDCS of bilateral cerebellum) [38] respectively. A large cathode was used to maintain the current density of the reference electrode at a low level, as such a reference electrode was demonstrated to be functionally ineffective without compromising the effectiveness of tDCS regarding the stimulation electrode [39,40]. The anatomical landmark for bilateral M1 (leg area) was determined according to previous studies which based their peak coordinates on Transcranial magnetic stimulation (TMS)-measurements [41,42,43]. For anodal tDCS of M1, we placed the anode 1 cm behind the vertex (Cz) on the mid-sagittal line to cover both leg motor cortices. For anodal cerebellar stimulation, the anode was placed 2 cm below the inion [38,44]. During SH-tDCS, a 2 mA current was maintained for 30 s before being ramped down and terminated. To improve the blinding procedure, M1-tDCS or CB-tDCS montages were randomly used as SH-tDCS montages. Before and after tDCS, participants rated their level of perceived sensation in relation to the stimulation (0 = no sensation, 10 = unbearable sensation) on a visual analog scale (VAS).

### 2.5. tDCS Current Flow Simulation

**Figure 2:**
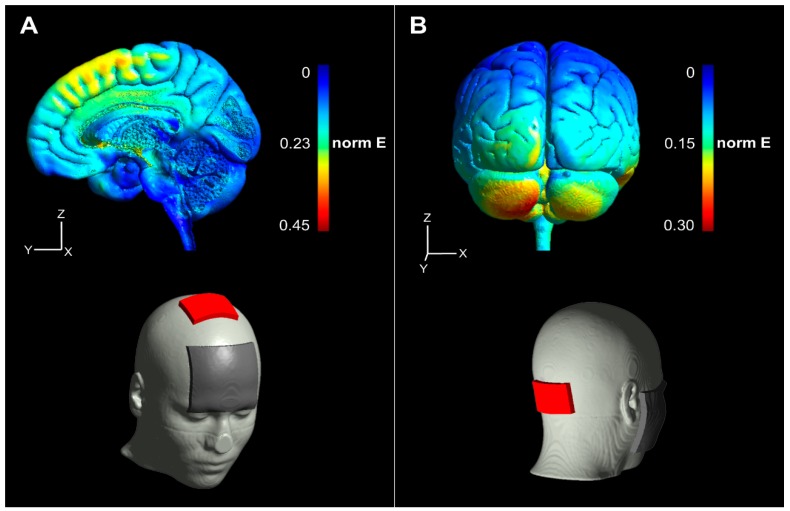
Transcranial direct current stimulation (tDCS) current flow simulation. Simulated current flow is illustrated for primary motor cortex (M1)-tDCS (**A**) as well as cerebellar (CB)-tDCS (**B**). Anodes are depicted as red rectangles and cathodes as gray rectangles projected on a standard head model in the lower half of (**A**) and (**B**), respectively. Normalized electrical field strength (V/m) is indicated through colormaps with blue representing lowest and red representing highest field strengths, respectively.

We simulated electric field distributions based on a finite element model of a representative head inside the open-source SimNIBS software (www.simnibs.org) to approximate current flow based on our tDCS configurations. For both M1-tDCS and CB-tDCS conditions, anodes, and cathodes were defined according to the above-mentioned positions. For both simulations, a current of 2 mA was selected. For anodal tDCS of M1 (leg area), the maximum electrical field strength (0.53 V/m) was determined below the anode, corresponding to the leg area of M1 with a posterior–anterior current flow direction towards premotor areas (Figure 2A). Anodal cerebellar tDCS electrical field strength was highest in the left cerebellar hemisphere (0.56 V/m) with posterior–anterior current flow direction towards brain stem areas (Figure 2B).

### 2.6. Data Analysis

**Figure 3:**
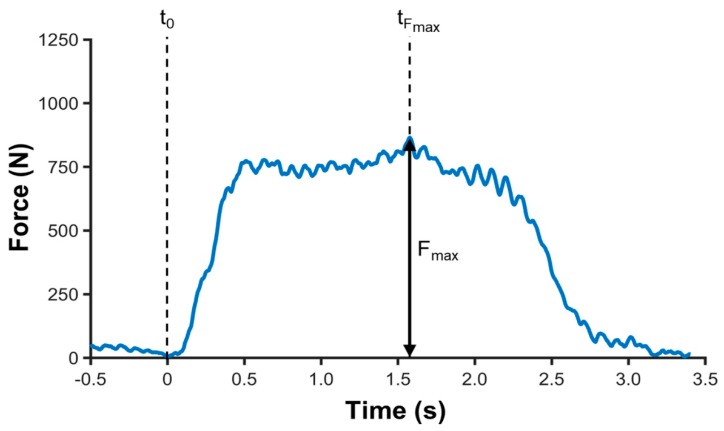
Determination of MIVC. Exemplary MIVC determination is illustrated on a single force–time curve. Force–time onset (t_0_), peak force value (F_max_), as well as time to peak force value (t_Fmax_) are highlighted.

Analyses were performed using customized MATLAB^®^ (v. R2019b, The MathWorks Inc., Natick, MA, USA) scripts. For each subject, data were evaluated thoroughly with incorrect measurements being excluded. Peak force values (N) were extracted out of each measurement and used for further analyses (cf., Figure 1B). Data were then normalized to individual baseline performances on each different session (M1-tDCS, CB-tDCS, SH-tDCS). For an exemplary depiction of MIVC determination, please see Figure 3. Normal distribution was assessed through Lilliefors-testing (α = 0.05). All data were subjected to repeated-measures analyses of variance (_rm_ANOVA) with stimulation (STIM) (M1-tDCS, CB-tDCS, and SH-tDCS) and measurement times (TIME) (before stimulation, 10, 15, and 20 during stimulation and 10 min after stimulation termination) as within-subject factors for the dependent variable MIVC to compare tDCS induced MIVC effects. Additionally, we compared online (during tDCS stimulation) and offline (after tDCS stimulation) effects of tDCS. For this purpose, we compared averaged MIVC values across timepoints during stimulation (10, 15, and 20) with MIVC values during POST. Potential sphericity violations were adjusted according to Greenhouse–Geisser (epsilon < 0.75) or Huynh–Feldt correction (epsilon > 0.75). Statistical thresholds were set at *p* < 0.05. Post hoc analyses were conducted by way of Bonferroni–Holm correction for multiple comparisons. To examine the effect of potential outliers, we used a common procedure to detect and remove outliers. First, we computed the mean and standard deviation (SD) of all MIVCs per participant and condition. Then, we excluded datasets, which fell more than 2.5 SD from the mean [45,46,47] and reconducted analyses to uncover potential differences in the obtained results. 

## 3. Results

We did not find significant differences regarding perceived stimulation sensation between sessions (F(2,38) = 0.222, *p* = 0.802, ηp^2^ = 0.001). None of our participants reported any tDCS related side effects and no participant reported discomfort during stimulation.

### MIVC During iBS

**Figure 4:**
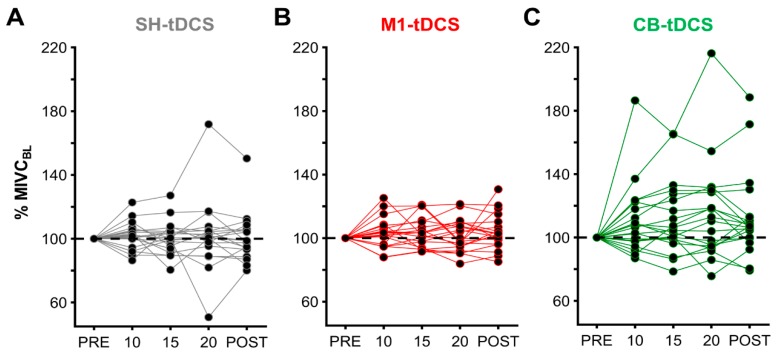
MIVC results I. Individual MIVC results illustrated as percentage-wise increase respective to baseline MIVC values (% MIVC_BL_), per sham (SH)-TDCS (**A**), M1-tDCS (**B**), and CB-tDCS (**C**) for all participants.

For an overview of all individual MIVC results per stimulation please see Figure 4.

**Figure 5:**
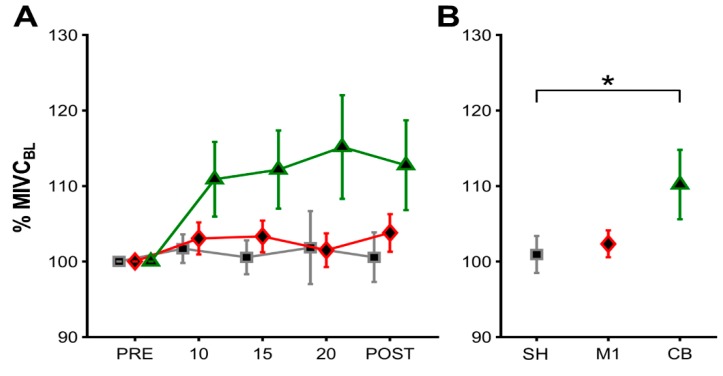
MIVC results II. Mean MIVC and standard error of the mean (SEM) values illustrated as percentage-wise increase respective to baseline MIVC values (% MIVC_BL_), per SH-TDCS (grey squares), M1-tDCS (red diamonds), and CB-tDCS (green triangles) for all participants. (**A**) Depicts mean MIVC results averaged across participants, while (**B**) illustrates mean MIVC results averaged across participants and time to highlight general tDCS effects. Asterisks indicate significant differences between tDCS stimulations. Respective *p* values are reported in the Results section.

Here, we found a significant main effect for STIM (F(2,38) = 4.369, *p* = 0.027, ηp^2^ = 0.187), with post-hoc Bonferroni-Holm tests revealing MIVC values to be higher for CB-tDCS when compared to SH-tDCS (*p* = 0.028) (Figure 5B), with no significant effects for CB-tDCS vs. M1-tDCS (*p* = 0.076), as well as M1-tDCS vs. SH-tDCS (*p* = 0.681). When averaging MIVC values across TIME per stimulation we found MIVC to increase by 1.16% for SH-tDCS and 2.91% for M1-tDCS as compared to 12.70% for CB-tDCS (Figure 5B). We did not find significant main effects for TIME (F(3,57) = 0.099, *p* = 0.005, ηp^2^ = 0.146) (Figure 5A), or a STIM*TIME interaction (F(6,114) = 0.608, *p* = 0.633, ηp^2^ = 0.031). Following the outlier detection, one dataset was removed, and all analyses were reconducted. However, the results did not change as we still observed an effect for STIM (F(2,36) = 4.101, *p* = 0.033, ηp^2^ = 0.186), with post-hoc Bonferroni–Holm tests revealing MIVC values to be higher for CB-tDCS when compared to SH-tDCS (*p* = 0.022), with no significant effects for CB-tDCS vs. M1-tDCS (*p* = 0.255), as well as M1-tDCS vs. SH-tDCS (*p* = 0.833). Accordingly, the outlier had no effect on our results and we therefore elected to display the results with all original data.

Lastly, we assessed differences in MIVC values averaged across timepoints during stimulation (10, 15, and 20) with MIVC values during POST to compare online and offline tDCS effects. We found a significant main effect for STIM (F(2,38) = 4.049, *p* = 0.025, ηp^2^ = 0.176), with post-hoc Bonferroni–Holm tests revealing MIVC values to be higher for CB-tDCS when compared to SH-tDCS (*p* = 0.033) with no significant effects for CB-tDCS vs. M1-tDCS (*p* = 0.109), as well as M1-tDCS vs. SH-tDCS (*p* = 0.613). We did not find significant main effects for online vs. offline tDCS effects on MIVC (F(1,19) = 0.011, *p* = 0.918, ηp^2^ = 0.001) or an interaction between both factors (F(2,38) = 0.367, *p* = 0.695, ηp^2^ = 0.019).

## 4. Discussion

In the present study, we evaluated the effects of anodal tDCS (M1-tDCS and CB-tDCS) on MIVC during iBS. We observed a significant increase in MIVC during iBS for CB-tDCS compared to SH-tDCS. To the best of our knowledge, the present study is the first to examine tDCS effects on muscle strength parameters during a whole-body movement. Additionally, we provide the first description of force enhancing effects during non-invasive cerebellar stimulation. Lastly, we did not find differences between online and offline MIVC enhancement. We believe that these results are of importance for several research fields as we provide an initial reference point and highlight potential challenges for future studies. 

The main result of this study is an increase in MIVC for CB-tDCS stimulation but no significant increase for M1-tDCS or SH-tDCS. On one hand, an increase in muscular force after tDCS administration is in line with previous studies [12,13,14,15], albeit excluding CB-tDCS, yet in contrast to other results that failed to find such effects on muscle strength parameters [16,17,18]. Several factors may account for this. 

First, it seems important to discuss our paradigm within the context of previous related research. Concerning this matter, we believe our tDCS paradigm to be consistent with most studies examining tDCS-induced muscle strength modulations [8]. Stimulation sites of both M1-tDCS, as well as CB-tDCS, were chosen based on common directives from recent literature [8,38,44]. We selected a current of 2 mA, as it has been reported that a current of 2 mA penetrates deeper through the skull when compared to 1.5 mA or 1 mA [48] and is commonly employed when administering tDCS over CB or M1. This might be a critical point considering an absent effect of M1-tDCS on MIVC, as M1 (leg area) is located deep inside the longitudinal cerebral fissure [49]. On the contrary, higher current intensity does not necessarily increase cortical excitability [50,51]. It has been suggested, that lower current intensities (<2 mA) are potentially more beneficial regarding tDCS effects, meaning that even if currents of higher intensity generally penetrate deeper through tissue, they do not always reflect optimal intensities to modulate local excitability [52]. Still, current flow simulations based on both tDCS configurations employed in our study show target areas (M1 and CB) to be extensively covered by the administered current (see Figure 2A,B). It is, therefore, conceivable that M1 plays a subordinate role in the force modulation of iBS. While both target areas are covered, current additionally spread broadly across other areas of the brain. This issue of low spatial focality is well known in tDCS research [8,13,52], although many studies show tDCS modulations of single muscle activity [48] and single muscle performance [12,14,53]. Additionally, concerning the effects of CB-tDCS on MIVC, it could be argued that due to our montage current is spreading beyond the cerebellum, specifically to regions of the brain stem and therefore, our results cannot be attributed to cerebellar modulations exclusively. However, a previous study assessed changes in brainstem excitability using the same CB-tDCS montage we employed in this study [54]. The authors did not find changes in brainstem excitability, which is why we assume such a confound to be unlikely regarding the present study. 

Considering the positive effect of CB-tDCS on iBS, it is important to contextualize the results with our task. The iBS task, as performed in the present study, represented a novel challenge to all participants as they were not skilled in iBS with a short familiarization serving as each individual’s only experience before testing. In this sense optimized coordination between muscles involved in force transmission during iBS is conceivable. Several studies show such adjustments as a result of CB-tDCS, e.g., during locomotor adaptation tasks [30] and complex overhand throwing tasks [55]. In particular, it should be mentioned that these studies that show such effects used protocols of anodal CB-tDCS in accordance with our results [30,55,56,57]. Another adaptation relating to coordination that could be induced by CB-tDCS is an optimized antagonist contraction behavior. Several studies have shown that increases in strength and improved force progression, following adaptation to strength training, are associated with reduced antagonist co-contraction [58,59]. It is also known that the cerebellum plays a crucial role in the coordination between agonist–antagonist contractions during exercise. For example, people suffering from cerebellar pathologies may experience increased co-contractions at the start of movement [60], inaccurate timing of antagonist muscles during agonistic contractions [60,61], and delayed onset of agonistic contractions [62]. Furthermore, studies show that the cerebellum contributes to the temporal coordination of movement patterns [61]. Interestingly, anodal stimulation of the cerebellum, combined with subsequent cerebral stimulation has been shown to improve the onset latency of antagonistic movements in patients with cerebellar ataxia [63]. Future studies should investigate the precise mode of action of CB-tDCS on antagonistic contractions and its relationship to improved strength performance in compound whole-body movements. In addition, adaptive motor control and the role of both areas of stimulation (M1-tDCS and CB-tDCS) during adaptive motor control potentially explain our results. In brief, adaptive motor control describes error reduction during motor adaptation [64]. Although both cerebellum and M1 are involved in motor control, their roles during adaptive motor control differ. Here, cerebellar processing is thought to affect online learning, evidenced through reductions in movement-related errors after CB-stimulation during motor tasks using tDCS [30,64,65] and TMS [66] stimulation. Further, cerebellar lesions lead to impaired abilities regarding successful changes in visuomotor positioning which further suggests that error reduction during motor tasks is a process connected to cerebellar activity [24,67]. On the other hand, M1-stimulation yields an offline response concerning adaptive motor control. This is reflected in previous research showing decreased retention time with no effects on error reduction after M1-stimulation [64,65]. These differences could help explain our finding that CB-tDCS increases MIVC during iBS. As CB-tDCS modifies cerebellar output, mainly that of Purkinje cells [54], it is possible that such a stimulation modulates cerebellar responses to sensory prediction error-related input and therefore leads to an increase in cerebellar response to task-related kinematic and dynamic errors [54,64]. Although this remains speculative, our results might, therefore, reflect an increase in the range of operating Purkinje cells as a response to CB-tDCS stimulation, leading to more rapid adaptations following each trial. This potentially results in greater force output, as timely coordination between segments (limbs and constrained body parts) in all joints is enhanced due to these rapid adaptational processes concerning the internal model of iBS. Therefore, we think that our results do not reflect greater force production capacity, but rather a quicker realization of force production due to improved coordination between muscles involved in iBS, possibly as a result of optimized internal models of iBS following CB-tDCS compared to M1-tDCS and SH-tDCS. 

Lastly, M1 is not likely involved in online motor control and force production during iBS. Still, previous studies showed force modulations of M1-tDCS [12,13,14], which is why the absence of such an effect in this study has to be addressed. An initial problem lies in the interindividual heterogeneity of M1 representations [68], inferring that current which sufficiently covers a targeted area and thus enhances local excitability in one participant might fail to do so entirely in another. This issue is closely related to overall variability in tDCS responsiveness. Generally, many participants fail to respond to tDCS stimulation expectedly [69,70]. In particular cases, nearly half of all stimulated participants do not respond to stimulation as anticipated [71]. Inter-individual variability in tDCS responsiveness has been observed for hand [69,70,71] and leg area of M1 [72,73]. TMS can be used as a countermeasure to ensure consideration of individualities concerning cortical representations. Interestingly, combining TMS-localization with tDCS stimulation does not guarantee responsiveness. Out of two studies that examined tDCS related muscle strength modulations in lower extremities and used TMS to localize and stimulate individual target areas, one reports high responsiveness and behavioral enhancement [14], whilst the second does not [18]. Still, future studies should consider using TMS, as it would have allowed for a definite control measure regarding the effectiveness of tDCS stimulations. High-definition tDCS (HD-tDCS) is another approach to increase the focality of non-invasively administered current. It was shown that HD-tDCS current is generally more spatially focal and able to increase the duration of tDCS induced after-effects compared to conventional tDCS [74]. Two studies have applied HD-tDCS to examine its effects on muscle strength parameters but were unable to find positive results [75,76]. Finally, a limitation of this study is the number of MIVC tests performed per measurement point. We have decided on one MIVC measurement per measurement point since the risk of injury to the participants is increased with each additional measurement per measurement point. We were recommended to do so by biomechanical experts during the evaluation of our test set-up. Since the squat, as a whole-body movement, demands the entire muscle apparatus, we wanted to avoid additional risks due to accumulated fatigue. In addition, the resting periods between several MIVCs, especially during whole-body movements, should be at least 3–5 min apart to ensure maximum force generation in each trial [77].

## 5. Conclusions

In conclusion, we provide novel evidence that anodal CB-tDCS can enhance MIVC in iBS serving as a whole-body movement. This is the first study to observe force enhancing effects of tDCS on whole-body movements as well as the first study to show force enhancing effects of CB-tDCS. As such, we present an initial framework of references concerning tDCS effects on muscle strength parameters in whole-body movements. A multitude of everyday life activities is comprised of whole-body movements. TDCS induced improvements of movement parameters, such as MIVC, in whole-body movements could prove beneficial to enable improved physical functioning, as well as prevent age-related decline in motor function. Perspectively, it seems important to test dynamic movements as whole-body movements commonly include both dynamic (eccentric and concentric) and static (isometric) movement periods. Therefore, it is necessary to extend our findings by including different protocols as well as examining other whole-body movements in future studies.
